# Mass spectrometric determination of early and advanced glycation in biology

**DOI:** 10.1007/s10719-016-9709-8

**Published:** 2016-07-20

**Authors:** Naila Rabbani, Amal Ashour, Paul J Thornalley

**Affiliations:** 1 Warwick Systems Biology Centre, Senate House, University of Warwick, Coventry, CV4 7AL UK; 2 Clinical Sciences Research Laboratories, Warwick Medical School, University Hospital, University of Warwick, Coventry, CV2 2DX UK

**Keywords:** Glycation, Mass spectrometry, Proteomics, Glucose, Methylglyoxal, fructosamine, Hydroimidazolone, Bioinformatics, Orbitrap

## Abstract

Protein glycation in biological systems occurs predominantly on lysine, arginine and N-terminal residues of proteins. Major quantitative glycation adducts are found at mean extents of modification of 1–5 mol percent of proteins. These are glucose-derived fructosamine on lysine and N-terminal residues of proteins, methylglyoxal-derived hydroimidazolone on arginine residues and N^ε^-carboxymethyl-lysine residues mainly formed by the oxidative degradation of fructosamine. Total glycation adducts of different types are quantified by stable isotopic dilution analysis liquid chromatography-tandem mass spectrometry (LC-MS/MS) in multiple reaction monitoring mode. Metabolism of glycated proteins is followed by LC-MS/MS of glycation free adducts as minor components of the amino acid metabolome. Glycated proteins and sites of modification within them – amino acid residues modified by the glycating agent moiety - are identified and quantified by label-free and stable isotope labelling with amino acids in cell culture (SILAC) high resolution mass spectrometry. Sites of glycation by glucose and methylglyoxal in selected proteins are listed. Key issues in applying proteomics techniques to analysis of glycated proteins are: (i) avoiding compromise of analysis by formation, loss and relocation of glycation adducts in pre-analytic processing; (ii) specificity of immunoaffinity enrichment procedures, (iii) maximizing protein sequence coverage in mass spectrometric analysis for detection of glycation sites, and (iv) development of bioinformatics tools for prediction of protein glycation sites. Protein glycation studies have important applications in biology, ageing and translational medicine – particularly on studies of obesity, diabetes, cardiovascular disease, renal failure, neurological disorders and cancer. Mass spectrometric analysis of glycated proteins has yet to find widespread use clinically. Future use in health screening, disease diagnosis and therapeutic monitoring, and drug and functional food development is expected. A protocol for high resolution mass spectrometry proteomics of glycated proteins is given.

## Protein glycation in biological systems

Protein glycation is a spontaneous post-translational modification (PTM) of proteins found in biological systems. It involves the non-enzymatic covalent attachment of a reducing sugar or sugar derivative to a protein [1]. It is a PTM that is often thermally and chemically labile when removed from the physiological setting, particularly at high pH and temperature. Analysis of protein glycation is compromised by use of heating and high pH in pre-analytic processing for mass spectrometric analysis [2].

**Fig. 1:**
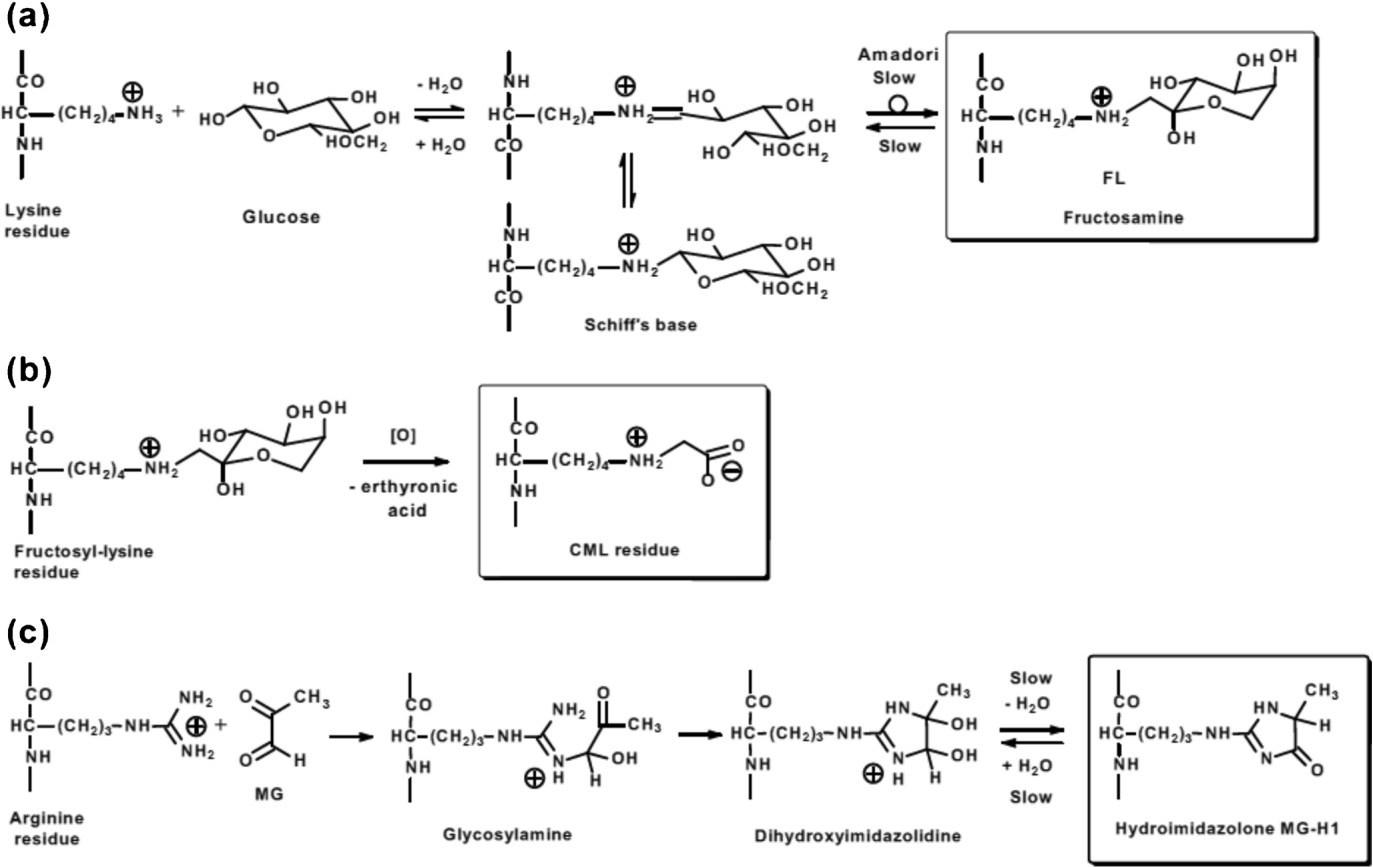
Major protein glycation processes in physiological systems. (**a**) Early glycation. Formation of the Schiff’s base and fructosamine (Amadori product) of lysine residues. (**b**) Oxidative degradation of fructosyl-lysine to N_ε_-carboxymethyl-lysine (CML). Similar processes occur on N-terminal amino acid residues. (**c**) Glycation of arginine residues by methylglyoxal with formation of dihydroxyimidazolidine and hydroimidazolone MG-H1 residues. There are related structural isomers and similar adducts formed from glyoxal and 3-deoxyglucosone [1, 3–5]

Glycation adducts are classified into two groups: early-stage glycation adducts and advanced glycation endproducts (AGEs). Glucose reacts with amino groups of lysine residue side chains and N-terminal amino acid residues to form sequentially a Schiff’s base and then, via the Amadori rearrangement, N^ε^-(1-deoxy-D-fructos-1-yl)lysine (FL) and *N*^α^-(1-deoxy-D-fructos-1-yl)amino acid residues – called collectively fructosamines – Fig.1a. FL is also known by synonyms fructosyl-lysine and fructoselysine. These are early-stage glycation adducts. Schiff’s base adducts are usually a minor component of glucose adducts *in situ*, *ca.* 10 % of the level of FL residues in the steady-state. They are also relatively rapidly reversed during sample isolation and processing, whereas fructosamines have much slower reversibility of formation; chemical relaxation times for reversal of Schiff’s base and fructosamine formation are *ca.* 2.5 h and 38 h at pH 7.4 and 37 °C, respectively [6, 7]. Accordingly when adducts of early-stage glycation by glucose are detected and quantified it is typically the fructosamine proteome that is characterised – as the Schiff’s base adduct reverses during protein isolation. Fructosamine modification of proteins is usually low, 5–10 mol% of the protein modified by one fructosamine residue. Examples of proteins found to be susceptible to fructosamine formation are given in Table 1. Collectively these and other proteins susceptible to fructosamine formation constitute the “fructosamine proteome”.

**Table 1:** Selected components of the fructosamine proteome

Species	Protein	Hotspot sites	Extent of modification	Functional impairment	Reference
Human	Apolipoprotein A1	K239	4 %	None	[8]
	Apolipoprotein E	K93	Unknown	Impairs heparin binding	[9]
	Bisphosphoglycerate mutase	K158	Unknown	Inactivation	[10]
	CD59	K41	Unknown	Inactivation	[11]
	Complement factor B	K266	Unknown		[12]
	Gastric inhibitory polypeptide	Y1	Unknown	Increased insulin release	[13]
	Glucagon-like peptide-1	1H	Unknown	Decreased insulin release	[14]
	Hemoglobin α_2_β_2_	α-K61, β-V1, β-K66	5 % (α:β, 0.6:1)	Increased oxygen binding in T state.	[15–17]
	Insulin	β-F1		Decreased activity.	[18]
	Microglobulin, β2-	I1	Unknown	Aggregation in chronic renal dialysis.	[19]
	Serum albumin	D1, K199, K439, K525	10 %	Decreased drug binding and leakage through the glomerular filter.	[20, 21]
	Superoxide dismutase-1	K122, K128	Unknown	Inactivation	[22]
Bovine	Crystallin, αA	K11, K78	Unknown		[23]
	Crystallin, αB	K90, K92	Unknown		[23]
	Crystallin, γB	G1, K2	Unknown		[24]
	Glutathione peroxidase-1	K117	Unknown	Inactivated.	[25]
	Insulin	α-G1, β-F1, β-K29	Unknown		[26]
	Major intrinsic peptide	K238, K259	Unknown	Affects membrane permeability.	[27]
	Ribonuclease A	K1, K7, K41	Unknown		[28]
	Serum albumin	K12, K136, K211, K232, K377, K524	10 %		[29]
Rat	Collagen-I	α1-K434	50–70 %	Increased susceptibility to cross-linking.	[30]
		α2-K453	27–33 %		
		α2-K479	24–29 %		
		α2-K924	22–28 %		
	Aldoketo reductase 1 A1	K67, K84, K140	18 %	Inactivation.	[31]

Extent of modification: data are for extent of modification *in vivo* of healthy subjects except for rat collagen which are percentage of total fructosamine adducts on each polypeptide chain (α1 and α2) from rat donors 6–36 months of age

In later stage reactions of glycation in physiological systems, Schiff’s base and fructosamine adducts degrade to many stable end-stage adducts or AGEs [1]. Endogenous α-oxoaldehyde metabolites are potent glycating agents and react with proteins to form AGEs directly. An important dicarbonyl glycating agent is methylglyoxal. The well-studied AGE, *N*^ε^-carboxymethyl-lysine (CML), is mainly formed by the oxidative degradation of fructosyl-lysine [7, 32] and hence has the same proteome site-specific coverage as the fructosyl-lysine precursor – Fig. 1b. There are also minor contributions to CML from glycation of proteins by glycolaldehyde and glyoxal, which may have a different site-specific distribution [33, 34]. A major AGE quantitatively found in biological systems is the methylglyoxal-derived hydroimidazolone, N_δ_-(5-hydro-5-methyl-4-imidazolon-2-yl)-ornithine (MG-H1) [3]. Comparisons of CML and MG-H1 residue contents of proteins, respectively, are (mean ± SD): healthy human subjects - plasma protein 0.038 ± 0.005 mmol/mol lys versus 0.31 ± 0.20 mmol/mol arg, and haemoglobin - 0.075 ± 0.023 mmol/mol lys versus 2.62 ± 0.60 mmol/mol arg (n – 12) [35]; and rats (Sprague Dawley, non-diabetic) – liver 0.100 ± 0.030 mmol/mol lys versus 3.34 ± 0.32 mmol/mol arg, skeletal muscle – 0.188 ± 0.093 mmol/mol lys versus 1.77 ± 0.77 mmol/mol arg, kidney (glomeruli) 0.27 ± 0.15 mmol/mol lys versus 2.42 ± 0.79 mmol/mol arg, brain 0.35 ± 0.07 mmol/mol lys versus 2.73 ± 0.39 mmol/mol arg, and plasma protein 0.042 ± 0.013 mmol/mol lys versus 1.23 ± 0.49 mmol/mol arg (*n* = 13) [3, 36, 37].

Methylglyoxal reacts predominantly with arginine residues to form sequentially a glycosylamine, dihydroxyimidazolidine and hydroimidazolone MG-H1 residues – Fig. 1c. Other structural isomers are also found: MG-H2 and MG-H3 [4]; isomer MG-H1 is usually dominant *in vivo* [38]. The half-life for reversal of glycosylamine/ dihydroxyimidazolidine formation is *ca.* 1.8 days and for reversal of hydroimidazolone is *ca.* 12 days at pH 7.4 and 37 °C [39]. The stability of the hydroimidazolone decreases with increasing pH; the half-life of MG-H1 is 0.87 days at pH 9.4 [4]. Hence both dihydroxyimidazolidine and hydroimidazolone residues derived from arginine residues may be detected in mass spectrometric analysis of glycated proteins. Glycation of proteins by methylglyoxal is found at levels of 1–5 mol% in most proteins but increases to *ca.* 50 % in the human lens of elderly subjects where there is limited protein turnover [5, 38]. It often occurs at functional domains of proteins and leads to protein inactivation and dysfunction. This may be because arginine residues have the highest probability (20 %) of any amino acid to be found in a functional domain and there is loss of positive charge on formation of MG-H1 [40, 41]; modification of these arginine residues outside functional domains of proteins is unlikely to lead to protein inactivation, unless involved in a key structural interaction – for example, ion-pair interaction R123 of human apolipoprotein A-1 [42]. Gene knockout of glyoxalase 1 (Glo-1), the enzyme that protects against glycation by methylglyoxal, is embryonically lethal and increased methylglyoxal concentration, or dicarbonyl stress, imposed by Glo-1 deficiency accelerates the ageing process and exacerbates diseases – including cardiovascular disease, diabetes, renal failure and neurological disorders [43]. Proteins susceptible to methylglyoxal glycation are called collectively the “dicarbonyl proteome”. Examples are given in Table 2.

**Table 2:** Selected components of the dicarbonyl proteome

Species	Protein	Hotspot sites	Arginine agent	Extent of modification	Functional impairment	Reference
Human	Apolipoprotein A1	R27, R123, R149	Methyl- glyoxal (MG)	*ca.* 1 %	R27 – increased catabolism; R123 – decreased stability; R149 – impaired functional activity.	[42]
	Apolipoprotein B100	R18	MG		Increased density, proteoglycan binding and atherogenicity	[44]
	Collagen-IV	α1-R390, α2-R889, α2-R1452, α3–1404	MG	*ca.* 5 %	Decreased integrin binding.	[45, 46]
	Crystallin, αA-	R12, R65, R157, R163	MG	Unknown	Increased chaperone activity	[47]
	ß-Defensin-2	R22, R23	Glyoxal /MG	Unknown	Decreased antimicrobial activity.	[48]
	Fibrin(ogen)	α-R167, α-R199, α-R491, α-R528, β-R149, β-R304	MG	Unknown	Abnormal thrombosis and fibrinolysis	[49]
	Heat shock protein-27	R75, R89, R94, R127, R136, R140, R188	MG	Unknown	Enhanced protection against oxidative stress.	[50]
	Hemoglobin α_2_β_2_	α-R31, α-92, α-141, β-R30, β-R 40, β-R104	MG	*ca.* 2.6 %	Increased oxygen binding	[35, 51, 52]
	HIF1α–co-activator p300	R354	MG	Unknown	Decreased hypoxia response	[53]
	Insulin	R46	MG	Unknown	Aggregation	[54]
	IgG (monoclonal)	LC-R30	MG	5 %	Acidic variant.	[55]
	Plasminogen	R504, R530, R561	MG	Unknown	Likely functional changes to cleavage and lys binding pocket in fibrinolysis.	[56]
	Proteasome, 20S subunits	β2-R85, β4-R224, β4–231, β5-123, β5–128	MG	Unknown	Decreased proteasome activity	[57]
Bovine	Ribonuclease A	R10, R39, R85	Glyoxal/MG	Unknown	Inhibition	[58, 59]
	Serum albumin	R114, R186, R218, R257, R410, R428	MG	*ca.* 1 %	Inhibition of esterase activity, prostaglandin breakdown and decreased drug binding.	[60, 61]
Mouse	mSin3a co-repressor	R925	MG	Unknown	Increased angiopoietin-2 activity	[62]

Extent of modification: data are for extent of modification *in vivo* of healthy subjects

Glycated proteins undergo proteolysis in physiological systems to release glycated amino acids called “glycation free adducts”. They are trace components of the amino acid metabolome. These are found in plasma and other body fluids. They are excreted from the body in urine. Urinary excretion of glycation free adduct increases from 2 to 15-fold in diabetes and renal failure [35, 63].

## Detection of total amounts of glycation adducts in multiplexed assay by liquid chromatography-tandem mass spectrometry

Some of the earliest applications of mass spectrometry to the study of glycated proteins was the detection of chemically-defined glycation adducts by gas chromatography-mass spectrometry. Prior acid hydrolysis and N- and O-acetylation of glycation adducts was required to produce low molecular mass volatile adducts suitable for detection [32]. The use of liquid chromatography-tandem mass spectrometry (LC-MS/MS) with an electrospray ionisation source avoided the requirement of chemical derivatisation of glycation adducts and in multiple reaction monitoring (MRM) data acquisition mode gave the high sensitivity and specificity of detection required to quantify glycation adduct in physiological systems. Use of Hypercarb™ graphitic chromatography retains glycation adducts during the chromatographic step to allow for diversion of non-volatile salts to waste before entry of analyte-containing eluate flow into the mass spectrometer. This minimises ion suppression and maintains a clean electrospray ionisation source for good, stable sample batch-to-batch performance [3]. Alternatively, ion-pair chromatography has been used [64]. Stable isotopic dilution analysis provides for robust quantitation and LC-MS/MS is now the analytical platform that dominates the field for robust, quantitative and multiplexed analysis of glycation adducts [64–66]. A wide range of glycation, oxidation and nitration adducts are routinely analysed by LC-MS/MS multiplexed assay in our laboratory. The protocol, detection conditions and analytical performance have been given elsewhere [65].

Stable isotopic dilution analysis LC-MS/MS may be applied for direct detection of glycation free adducts in ultrafiltrate of physiological fluids. The LC-MS/MS analysis is extended to quantify total glycation adduct contents of purified proteins and protein extracts of cells and extracellular matrix by prior exhaustive enzymatic hydrolysis [3]. The enzymatic hydrolysis method is similar to that employed by Henle *et al*. [67] with sequential addition of pepsin, pronase E and finally, added together, aminopeptidase and prolidase. We have made multiple modifications for improvement and specific applications: (i) aseptic processing with a sample autoprocessor (CTC-PAL, CTC Analytics, Zwingen, Switzerland) and inclusion of antibiotics after the acidic pepsin step to minimise bacterial contamination; and (ii) a modified procedure for specific proteins - using collagenase instead of pepsin for analysis of collagen [45], omitting pepsin for apolipoprotein B100 as some pepsin fragments are insoluble and resist further digestion [68], and performing the enzymatic hydrolysis under carbon monoxide for hemoglobin or red blood cell lysates to inactivate heme and prevent artefactual heme-catalysed glycoxidation [3, 35]. Analytical recoveries are *ca.* 90 % or higher for minimally glycated proteins but are lower in highly glycated proteins where there is resistance to proteolysis [3, 69]. An alternative method uses pronase E, aminopeptidase and carboxypeptidase Y and gave lower analytical recovery in the application studied [70]. Conventional acid hydrolysis cannot be used for acid labile AGEs such as hydroimidazolones for which very low analytical recoveries were found – *ca.* 10 % [4]. Acid hydrolysis may be used for acid-stable AGEs [71].

Acid hydrolysis has also been used for many years in the N_ε_-(2-furoylmethyl)lysine or furosine-based measurement of FL. The conversion of FL to furosine in acid hydrolysis is 32 %. Furosine is also formed from other Amadori products in food [72]. Recently a LC-MS/MS method has been developed for concurrent quantitation of furosine and acid-stable AGEs, CML and N_ε_-(1-carboxyethyl)lysine (CEL) [73].

## Methodological considerations for application of mass spectrometric proteomics to glycation research – glycated protein detection and quantitation

Proteomics studies provide a powerful approach to identify proteins susceptible to glycation in complex protein mixtures and also identify the lysine and arginine residues within proteins particularly susceptible to glycation. A typical workflow involves: (i) preparation of a protein extracts of samples of interest, (ii) reduction and alkylation of sample protein, (iii) limited proteolysis of proteins – usually by trypsin or lys-C and trypsin sequentially; (iv) partial resolution of tryptic peptides by nanoflow reversed phase liquid chromatography, and (v) detection and sequencing of tryptic peptides by high resolution mass spectrometry. Peptides are sequenced by fragmentation by collision induced dissociation (CID), high-energy collisional dissociation (HCD) or electron transfer dissociation (ETD) and detection and analysis of characteristic fragment ion series.

In all methods for proteomics studies, a critical requirement is unambiguous identification of proteins of interest. For many years a consensus criterion for protein identification was detection and sequencing of a minimum of two tryptic peptides unique in sequence, “unique peptides”, for the protein of interest. With the latest high resolution mass analysers this criterion has now been challenged and one unique peptide with estimation of false discovery rate is gaining acceptance as a criterion for protein identification [74].

Quantitation of tryptic peptides and thereby sample content of related proteins is an on-going challenge in proteomics studies. For analysis of complex protein mixtures quantitation is preferably based on mass spectrometric detection – rather than densitometry of spots of stained gels from 2-dimensional gel electrophoresis. This is because of potential interferences when using gel electrophoretic separation is the only basis for protein resolution. Nano-flow liquid chromatography-high resolution mass spectrometry is the major platform currently used for proteomics studies. A popular operating format is tryptic peptide molecular ion determination by ultrahigh resolution Orbitrap™ mass analyser and rapid consecutive peptide fragmentation for sequencing performed by an on-line ion trap mass analyser. For quantitation of proteins there are label-free and heavy isotopic labelling of sample methods. The most robust method of quantitation is use of stable isotope labelling with amino acids in cell culture (SILAC) and similar stable isotope labelling of mice. Stable isotopic lysine and arginine, [^13^C_6_]lysine and [^13^C_6_]arginine, are used in cell cultures to label proteins or in animal diets to label mouse tissue protein. Labelled cell culture reagents and mouse tissues are available commercially. Stable isotopic-labelled and normal, natural isotopic abundance, samples are processed identically and cell lysates or tissue extracts mixed prior to tryptic digestion to provide ^13^C-labelled internal standards for all peptides in subsequent stable isotopic dilution analysis work flow [75]. An alternative method is the introduction of isobaric tags for relative and absolute quantification (iTRAQ), which uses *N*-hydroxysuccinimide chemistry and *N*-methyl piperazine reporter group stable isotopic labels [76]. This may pose problems in glycation research for dicarbonyl proteome analysis as previous studies have shown the dicarbonyl moiety of hydroimidazolones migrates between arginine residues during *N*-hydroxysuccinimide active ester derivatisation conditions – see below. The iTRAQ protocol may require validation for glycation adduct detection and quantitation. A generally available method is label-free quantitation, which requires no additional sample manipulation but rather employs peptide ion responses for quantitation. Initially a normalization procedure for sample total ion current is performed using algorithm-based peak selection and exclusion so that only invariant ion responses are used in the normalization correction. The amount of tryptic peptide is then deduced from the sum of ion intensities of multiply-charged ion series of the peptide. For detection of an unmodified protein, the average, total or 3 most intense molecular ion intensities for unique peptides is used. Several commercial software tools are available for this analysis [77]. We have used Progenesis™ (Nonlinear Dynamics Ltd., Newcastle upon Tyne, U.K.) and Scaffold™ (Proteome Software, Inc., Portland, USA).

For application to detection and quantitation of glycated proteins, the glycated protein and its unglycated counterpart (typically 20–100 fold more abundant) are detected based on the ion intensities of the glycated and related unglycated tryptic peptide. This is only secure if the site of glycation is in a unique peptide; if not, the glycated peptide ion intensity has contributions of unknown proportions from different proteins. Often with glycation, a tryptic cleavage is missed and then the precursor dipeptide is preferably a unique dipeptide for secure identification of the related glycation proteins.

The methods for protein quantitation above provide relative quantitation of analytes. If absolute quantitation is required then the response is compared to that of analyte calibration standards and absolute amounts may be deduced. A further current development is LC-MS/MS with MRM analysis of glycated peptides after trypsinisation for absolute quantitation of particular glycated proteins in clinical and other samples [78, 79]. In this application, it is recommended that quantitation is based on a minimum of three molecular ion > fragment ion MRM transitions [80].

A great challenge for global screening of glycated proteins is to maximize sequence coverage of proteins in mass spectrometric analysis. Leading research teams performing total proteome analysis report a typical median sequence coverage of *ca.* 20 % [81]. A contributory factor to this is production of short peptides of ambiguous protein origin. This may be improved in some glycated proteins where the glycation adduct causes missed cleavage with trypsin and lys-C with resultant longer peptides. A recent computational approach has indicated that with judicious use of proteases the sequence coverage in proteomics analysis may increase to *ca.* 90 % [82]. Until this is routinely implemented, only a minor proportion of glycated proteins are likely detected and identified proteomics analysis.

## Bioinformatics

Bioinformatics tools for protein glycation are poorly developed. A particularly useful bioinformatics tool for glycation researchers would be a sequence search engine to predict sites susceptible to glycation in proteins. This can be approached empirically – building up a peptide motif glycation site motif on the basis of frequency of occurrence of amino acids on N- terminal and C-terminal sides of the target lysine or arginine residue glycated. For a non-enzymatic process such as glycation, a peptide susceptibility motif may also be predicted from a physicochemical approach where characteristics that make a particular lysine or arginine residue reactive towards glycation are considered. It is not yet possible to predict preferred, hotspot sites of protein glycation with surety. Current empirical data and physicochemical and glycation adduct turnover considerations are now described.

An examination of protein motifs for glucose glycation forming FL residues was made empirically by compiling and combining peptide motifs from published peptide mapping studies. It was found that K and R residues dominate in the N-terminal region and D and E residues dominate in the C-terminal region of FL sites but no clear motif for FL formation was found [83]. In a study of human plasma and red blood cells, detection and filtering for unique peptides with ≥5 spectrum counts gave 361 and 443 unique glycated peptide sequences from native human plasma and red blood cells, respectively. There was only limited evidence to support the hypothesis of N-terminal enrichment of K and R residues and C-terminal enrichment of D and E residues in the sequence motif for hotspot glycation by glucose [84].

**Fig. 2:**
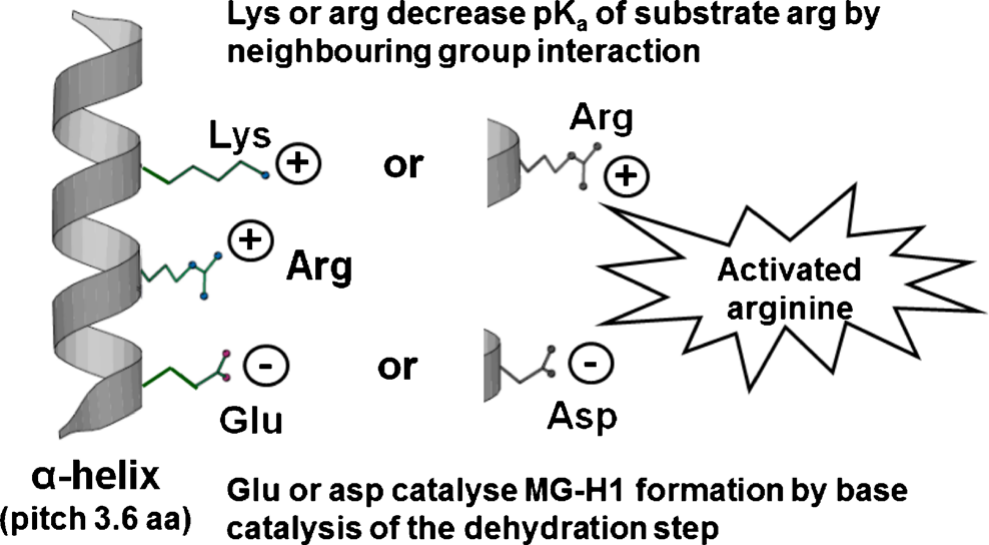
Activation of arginine residues in alpha-helix domains of proteins by neighbouring group interactions with basic and acidic amino acid residues. Figure reproduced with permission from [5]

Regarding physiochemical considerations, glycation is a non-enzymatic process and so selectivity for sites of glycation is determined by the reactivity of the lysine, arginine of N-terminal residue under consideration. This is linked to: (i) microscopic pK_a_ of the residue being modified, (ii) surface exposure of the modification site, and (iii) a proximate conjugate base catalyzing the dehydration step involved in FL and MG-H1 residue formation – Fig. 2.

Microscopic pK_a_ values of lysine, N-terminal and arginine residues have a profound influence on the site of glycation by glucose on N-terminal and lysine residues and on glycation by methylglyoxal of arginine residues. Microscopic pK_a_ values may be computed for proteins of known crystal structure – for example, by using the H++ automated system (http://biophysics.cs.vt.edu/H++) [85]. There is marked diversity of pK_a_ values of lys and arg residues in proteins. For example, in human serum albumin (HSA) microscopic pK_a_ values of the 59 lys residues vary from 7.9–14.0 and of the 24 arg residues vary from 12.2–18.6; an expected reactivity range of >10^6^; cf. reactivity of N-terminal serine pK_a_ of 7.9 [86]. The major sites of glycation by glucose in HSA are, in order of reactivity: N-terminal D1, K525, K199 and K439 [20]; cf. their rank order by increasing pK_a_ value of lysine side chain and N-terminal amino groups of first-equal, sixteenth, third and fourteeth. Low pK_a_ values are likely driving glycation of D1 and K199. Activating features of K525 and K439 may be deprotonation catalyzed by proximate E520/R521 and E442, respectively.

In a study of the hotspots sites of glycation of HSA by methylglyoxal, 3 of the 5 sites with MG-H1 residue formation had the lowest microscopic predicted pK_a_ values: R218, pK_a_ = 12.2; and R186 and R410, pK_a_ = 12.5. However, the remaining 2 sites, R114 and R428 with predicted pK_a_ values of 13.6 and 15.1, ranked 8th and 14th of 24 arginine residues in order of increasing microscopic predicted pK_a_ value. R114 has high surface exposure which likely also facilitates methylglyoxal modification. All activated arginine residues have a positively charged R or K residue 3 or 4 residues further along in the sequence that likely decreases the microscopic pK_a_ value and R428 only has a negatively charged residue, E425, preceding in the sequence. A subsequent study confirmed these hotspot sites except for R114 and suggested R257 as a further hotspot modification site, which has a relatively low pK_a_ (= 12.9) [60]. The proximity of a negatively charged D or E residue provides a conjugate base to promote the rate limiting removal of a proton from the protein-glucose Schiff’s base and arginyl-dihydroxyimidazolidine precursors of fructosamine and MG-H1 adducts, respectively. The combination of proximate cationic and anion side chain residues for lysine and arginine residue activation was initially proposed to explain site specificity of lysine residue glycation by glucose [87] and then applied to MG-H1 formation from arginine [5].

The above considerations are features relating to the rate of formation of glycation adducts. FL and MG-H1 residues have half-lives of *ca.* 25 and 12 days, respectively [4, 7], which exceeds the half-lives of most human proteins (median half-life 1.9 days [88]). Therefore, for many proteins the steady-state extent of protein glycation is also influenced by the half-life of the protein. Hence, early studies found the extent of glycation by glucose of several proteins *in vivo* was linked to the protein half-life [89]. Glycation leads to protein distortion and misfolding, as indicated by crystallographic studies of HSA glycated by glucose [90] and molecular graphics structural predictions for glycation of HSA, apolipoprotein B100 and apolipoprotein A-1 by methylglyoxal [42, 44, 61]. It is also expected that glycated proteins are targeted for cellular proteolysis and have an unusually decreased half-life. This remains to be determined in robust unfocussed, proteome dynamics studies where half-lives of unique tryptic peptides (which can be unambiguously linked back to particular proteins) and their glycated counterparts are determined in the same cell population. The level of FL and N-terminal fructosamine residues in cellular proteins is also influenced by enzymatic removal and repair by fructosamine-3-phosphokinase (F3PK) [91]. F3PK has different specific activity for FL residues in different sites in proteins. The FL residues detected at different sites in proteins are, therefore, a balance of the intrinsic reactivity for glycation and the reactivity of the FL residue site for repair by FP3K – see glycated haemoglobin, for example [92]. There is no known enzymatic mechanism for repair of MG-H1 residues.

## Pre-analytic processing and analytical protocols for mass spectrometric applications in glycation research

We gave updated pre-analytic processing protocols for detection of total amounts of glycation adducts in multiplexed assay by LC-MS/MS recently [65]. We give herein a similar protocol for detection of glycated proteins by high resolution mass spectrometry proteomics – Table 3.

**Table 3:** Protocol for high resolution mass spectrometry proteomics of glycated proteins

Step	Description	Procedure
1	Preparation of biological samples	Prepare fractional proteome cell extract as for analysis of total glycation adduct content *e.g*. for proteins >10 kDa molecular mass of the cytosolic proteome, cells (*ca.* 1 x 10^6^) are lysed by sonication in 10 mM sodium phosphate buffer, pH 7.4 and 4 °C, and membranes sedimented by centrifugation (20,000 g, 30 min, 4 °C). The supernatant is removed and washed by 5 cycles of concentration and dilution in water over a 10 kDa microspin ultrafilter. Protein is finally concentrated and assayed by the Bradford method.
2	Alkylation	To an aliquot of cytosolic protein extract (100 μg, 20 μl), dithiothreitol (6 μl, 6 mM) is added and the sample incubated at 37 °C in the dark for 30 min. Iodoacetamide solution (5.9 μl, 10.8 mM) is then added and the sample incubated at 37 °C in the dark for 30 min. Residual iodoacetamide is quenched by further addition of dithiothreitol (5.9 μl, 6 mM) and incubated at 37 °C in the dark for 30 min. An aliquot of Lys-C protease (1 mg/ml, 5 μl) in 500 mM ammonium bicarbonate, pH 8.0, is added and incubated for 1 h at 37 °C. Then tosyl phenylalanyl chloromethyl ketone (TPCK)-treated trypsin (1 mg/ml, 5 μl) in 1 mM calcium chloride/500 mM ammonium bicarbonate, pH 8.0, is added and samples were incubated at 37 °C for 5 h in the dark. The sample is then lyophilised to dryness and re-suspended in an aliquot (100 μl) 0.1 % formic acid in water and analysed by nanoflow liquid-chromatography-Orbitrap mass spectrometry.
3	Peptide separation, protein identification and quantitation	An aliquot of sample (5 μl) is injected and peptides partially resolved by nanoflow capillary liquid chromatography – see footnote. Peptides were eluted directly (300 nl min^−1^) via a Triversa Nanomate nanospray source (Advion Biosciences, NY, USA) into a Thermo Orbitrap Fusion (Q-OT-qIT, Thermo Scientific) mass spectrometer. Survey scans of peptide precursors from 350 to 1500 *m*/*z* are performed at 120 K resolution (at 200 *m*/*z*) with automatic gain control (AGC) 4 × 10^5^. Precursor ions with charge state 2–7 were isolated (isolation at 1.6 Th in the quadrupole) and subjected to HCD fragmentation. HCD was programmed to 35 % and used for rapid scan MS analysis in the ion trap where AGC is set to 1 x 10^4^ and the maximum injection time was 200 ms. Dynamic exclusion duration was set to 45 s with a 10 ppm tolerance around the selected precursor and its isotopes. Monoisotopic precursor selection is turned on. The instrument was run in top speed mode with 2 s cycles.
4	Data collection	Sequence information from the MS/MS data was managed by converting the raw (.raw) files into a merged file (.mgf) using MSConvert in ProteoWizard Toolkit (version 3.0.5759) [93] The resulting. Mgf files were searched, and the database was searched against protein sequence databases.
5	Data Analysis	Database search MS^2^ spectra are searched with Mascot engine (Matrix Science, version 2.5.0) against *Homo sapiens* database (http://www.uniprot.org/) assuming trypsin digestion. To determine false-positive peptide identifications, spectra are also searched against the corresponding reverse database, common Repository of Adventitious Proteins Database (http://www.thegpm.org/cRAP/index.html). Search parameters for Precursor mass and product ions tolerance are, respectively, ± 5 ppm and ±0.8 Da, allowing for two missed trypsin cleavages, cysteine carbamidomethylation and methionine oxidation. Only fully tryptic peptide matches were allowed.
6	Validation	Scaffold (version Scaffold_4.3.2, Proteome Software Inc.) is used to validate MS/MS based peptide and protein identifications from MS/MS sequencing results. Peptide identifications are accepted if established at > 95.0 % probability by the Scaffold Local FDR algorithm. Protein identifications were accepted if established at >95.0 % probability and contained at least 3 identified peptides – two of which are unique. Protein probabilities were assigned by the Protein Prophet algorithm [94]. Proteins that contained similar peptides and could not be differentiated based on MS/MS analysis alone were grouped to satisfy the principles of parsimony. Proteins sharing significant peptide evidence were grouped into clusters.

Footnotes: Instrumentation and **c** hromatography: Reversed phase nanoflow liquid chromatography- mass spectrometry for global protein identification is performed on an Orbitrap Fusion (Thermo) mass spectrometer equipped with a microspray source operating in positive ion mode. The column used is: an Acclaim PepMap μ-pre-column cartridge (trap), 300 μm i.d. × 5 mm, 5 μm particle size, 100 Å pore size, fitted to an Acclaim PepMap RSLC 75 μm i.d. × 50 cm, 2 μm particle size, 100 Å pore size main column (Thermo). It was installed on an Ultimate 3000 RSLC nano system (Tthermo). The peptides are eluted off of the trap onto the analytical column. Mobile phases were: A - 0.1 % formic acid in water, and B - 0.1 % formic acid in acetonitrile. The flow rate was programmed at 0.3 μl/min. Mobile phase B was increased from 3 % to 35 % in 125 to 220 min (depending on the complexity of the sample). Mobile phase B was then increased from 35 % to 80 % in 5 min before being brought back quickly to 3 % in 1 min. The column was equilibrated at 3 % of mobile phase B for 15 min before the next sample

Statistical analysis: The mean, standard deviation, confidence score and ANOVA test for all proteins are determined using datasets of a minimum of 3 independent sample digests using bioinformatics sand statistical analysis by Progenesis QI for proteomics 2.0 (Nonlinear Dynamics). Protein and peptide identification probabilities are performed using Progenesis

## Example of application of mass spectrometry in studies of early glycation adducts: fructosamine

### Fructosamine residues in peptides and proteins

The mass increment indicating the detection of FL and other fructosamine-modified peptides is +162 Da. Glycation of intact proteins and large peptide chains has been detected by electrospray positive ion mass spectrometry and matrix-assisted laser desorption-time of flight (MALDI-TOF) mass spectrometry. Roberts and co-workers detected and quantified fructosamine modified α- and β-chains of haemoglobin by deconvolution of multiply charged ion series [95], shown in later studies by peptide mapping to reflect fructosamine formation at sites α-K61, β-V1 and β-K66 [15]. Increase in molecular mass of HSA glycated by glucose prepared *in vitro* was measured by MALDI-TOF. This revealed that preparations of glycated HSA had a large increase in mass due to high extent of glycation, dissimilar from the low increase in mass of glycated HSA in plasma samples *in vivo*. For example, HSA from human plasma had mean mass increment of +243 Da, whereas model glucose-modified albumin prepared *in vitro* had a mean mass increment of +6780 Da [96]. This suggested the albumin prepared with very high extent of glycation was a poor model for the albumin with minimal extent of glycation found *in vivo*.

**Fig. 3:**
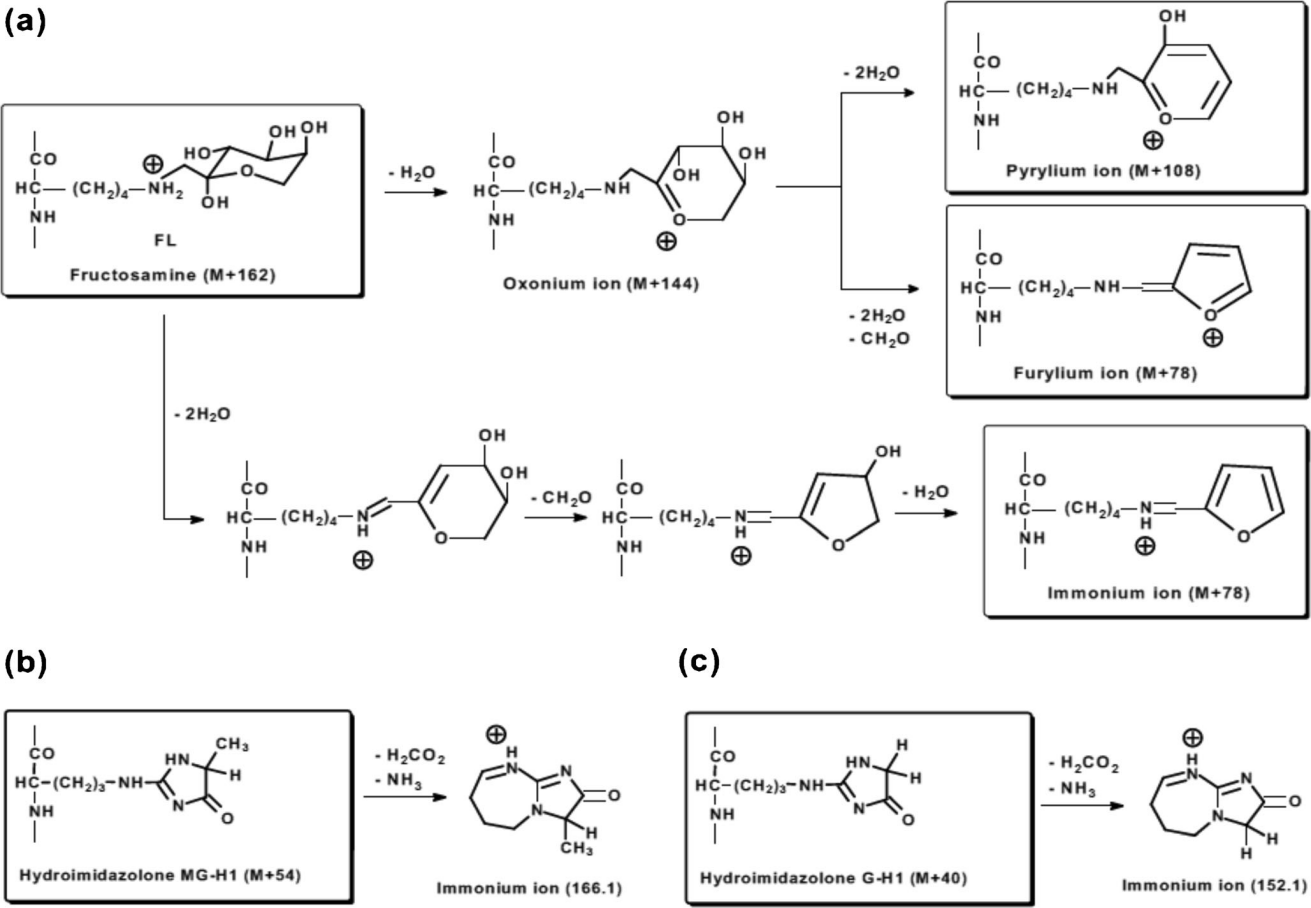
Fragmentation of fructosamine and hydroimidazolone glycation adducts. (**a**) Fragmentation of fructosyl-lysine by CID leading to formation of oxonium, pyrylium, furylium and immonium ions. (**b**) and (**c**) fragmentation of hydroimidazolones formed by methylglyoxal and glyoxal to immonium ions in CID and HCD [29, 97–99]

For mass spectrometric analysis of glycated peptides, CID and HCD fragmentation of fructosamine-containing peptides produced characteristic fragment ions of the precursor fructosamines (M + 162): by dehydration to an oxonium ion (M + 144), further dehydration to a pyrylium ion (M + 108), and dehydration and formaldehyde loss immonium ion (M + 78) [29, 97–99] – Fig. 3a. Pyrylium and furylium ions are detected in *y* ion series providing for fructosamine location [29]. In ETD fragmentation abundant and almost complete series of c- and z-type ions were observed, which greatly facilitated the peptide sequencing and fructosamine site location [100].

The FL degradation product, CML, is detected at the same sites as fructosamines residues in serum albumin, haemoglobin and ribonuclease A [29, 101, 102].

### Enrichment strategies of fructosamine-modified proteins

A boronate affinity chromatography enrichment method has been used to facilitate detection of the fructosamine proteome based on the binding of the *cis*-1,2-diol structure of fructosamine-modified proteins, with subsequent release from the boronate affinity matrix with weak acid. Although some enzymatically glycosylated proteins contain *cis*-1,2-diol moieties, steric effects, proximate negatively charged groups and acetylation limit the retention and interference in this method by glycoproteins [103]. A similar affinity method is used in the routine separation of hemoglobin in clinical chemistry to quantify glycated hemoglobin HbA_1c_ for assessment of glycaemic control in diabetes [104].

In principle antibodies to fructosamine may be used for immunoaffinity purification and enrichment of proteins glycated by glucose. Fructosamine may be reduced to hexitol-lysine residues prior to enrichment and then immunoaffinity purified with anti-hexitol-lysine antibody [105]. Where immunoaffinity enrichment is employed it is vital to confirm the presence of a glycation adduct residue or hexitol-lysine following reductive pre-analytic processing in the retained proteins by mass spectrometric analysis.

### Global analysis of fructosamine-modified protein

For the fructosamine proteome, phenylboronate affinity chromatography was used to enrich glycated proteins and glycated tryptic peptides from both human plasma and erythrocyte membranes. Enriched proteins are processed for limited proteolysis by trypsin or trypsin and lys-C. Trypsinisation cleavage after lysine residues is impaired by glycation by glucose and glycated peptide with missed-cleavage at the glycation site is detected [84].

The rate of fructosamine degradation at 37 °C increases markedly above pH 8 through increased reversal of the Amadori rearrangement and oxidative degradation to CML and related N^α^-carboxymethyl-amino acids [106, 107] – Fig. 1b. Use of high pH and high temperatures in pre-analytic processing of samples in glycation studies are avoided to maintain sample glycation analyte integrity. Conventional tryptic digestion in proteomics studies often uses tryptic digestion for 24 h at pH 8.5 with heating at 50 – 60 °C in some protocols [108]. This is preferably avoided in glycation studies where tryptic digestion at pH 7.4 and 37 °C is preferred.

Analysis of tryptic digests by liquid chromatography-tandem mass spectrometry with ETD peptide fragmentation identified 76 and 31 proteins with fructosamine modification from human plasma and erythrocyte membranes, respectively. The ETD fragmentation mode enabled identification of a higher number of glycated peptides (88 % of all identified peptides) compared to CID mode (17 % of all identified peptides) for samples enriched on protein and peptide levels [103]. In other studies with boronate affinity enrichment of proteins in human plasma and red blood cells, 7749 unique glycated peptides corresponding to 3742 unique glycated proteins were identified [84].

## Example of application of mass spectrometry in studies of advanced glycation adducts: methylglyoxal-derived hydroimidazolone and dihydroxyimidazolidine

### Methylglyoxal-derived hydroimidazolone and dihydroxyimidazolidine in peptides and proteins

Methylglyoxal-derived hydroimidazolone and dihydroxyimidazolidine may be detected in peptides glycated by methylglyoxal and tryptic peptides of proteins glycated *in vivo*. They have mass increments on arginine residues of +54 Da and +72 Da, respectively. A further minor methylglyoxal-derived and stable AGE, CEL, may be detected as +72 Da on lysine residues [42, 44, 58, 61]. High collision energy fragmentation may dehydrate dihydroxyimidazolidine to hydroimidazolone and so discrimination is provided by detection of the peptide molecular ion [58].

In analysis of methylglyoxal-modified lipoproteins no advantage of ETD over CID fragmentation in the detection of hydroimidazolone and dihydroxyimidazolidine was found [42, 44]. Fragmentation of peptides modified by MG-H1 and related isomers gave complete series of *b* and *y* ions with mass increment of 54 Da relative to those of unmodified peptide and no neutral losses [42, 44, 58, 61]. A MG-H1-related, peptide free side chain fragment ion of m/z = 166.1 can be observed in the low-mass region of the MS/MS spectra, with proposed immonium ion structure – Fig. 2b. A similar peptide-free side-chain fragment ion of m/z = 152.1 can be observed for glyoxal-modified peptides [109] – Fig. 2c.

Hydroimidazolone and dihydroxyimidazolidine residues are chemically labile AGEs and conditions of pre-analytic processing for proteomics analysis may influence mass spectrometric analysis outcomes. Tryptic digestion methods with prolonged periods of sample incubation at high pH and/or temperature leads to reversal of hydroimidazolone to dihydroxyimidazolidine and de-glycation. Alternatively, high pH and temperature may also stimulate dicarbonyl formation [110]. In earlier studies using *N*-hydroxysuccinimidyl active ester derivatisation of MG-H1 in chromatographic analysis, we found that incubation of MG-H1 in the presence of [^15^N_2_]arginine at pH 8.8 for 10 min at 55 °C led to migration of the methylglyoxal moiety from MG-H1 to [^15^N_2_]arginine [4]. Hence, use of high pH and temperature in pre-analytic processing may induce migration of the methylglyoxal moiety between arginine residues and, potentially, also between proteins. Conventional tryptic digestion techniques require modification to minimise increase of pH and avoid sample heating for peptide mapping and proteomics analysis of methylglyoxal-modified proteins and related PTMs.

Trypsin cleavage after arginine residues is impaired by glycation by methylglyoxal and glycated dipeptides with missed-cleavage at the glycation site are detected [60, 61]. In some cases, cleavage after dicarbonyl glycation of arginine was observed [109].

### Enrichment strategies for methylglyoxal-modified proteins

The dihydroxyimidazolidine residues present in proteins glycated by methylglyoxal and glyoxal are also a potential interference in boronate affinity chromatography for enrichment of fructosamine-modified proteins as they contain a side chain with a *cis*-1,2-diol moiety [111]. This has been exploited to identify proteins with arginine residues activated for reaction with glyoxal derivatives. Reaction of proteins with butan-2,3-dione formed 4,5-dihydroxy-4,5-dimethylimidazolidine residues of proteins containing activated arginine residues. Proteins with such residues on the surface were retained in boronate affinity chromatography [112, 113]. Antibodies to hydroimidazolones may be used for immunoaffinity purification and enrichment of proteins glycated by methylglyoxal. The anti-MG-H1 monoclonal antibody IG7 has been widely used for immunoblotting of MG-H1 and would be suitable for this application [114]. Where employed it is vital to confirm the presence of hydroimidazolone residues in the retained proteins by mass spectrometric analysis [2].

### Global analysis of methylglyoxal-modified proteins

In a recent report [109] plasma digests were analysed by nanoflow chromatography-LTQ Orbitrap XL ETD mass spectrometry and tryptic peptides scanned for m/z 152.1 and 166.1 side chain fragment ions indicative of glyoxal- and methylglyoxal-modified peptides – see above and Fig. 2b and c. Forty-four peptides representing 42 proteins were annotated. Arginine modifications were mostly represented by glyoxal-derived hydroimidazolones (34 peptides/39 sites) and methylglyoxal-derived dihydroxyimidazolidine (8 peptides/8 sites) and MG-H1 (14 peptides/14 sites). Use of high temperature and pH processing in this study may have compromised the outcome; many glyoxal modified proteins were detected whereas LC-MS/MS analysis typically shows very low amounts of glyoxal-derived-AGEs, hydroimidazolone and N_ω_-carboxymethylarginine, in plasma protein [35].

In pilot studies using nanoflow liquid chromatography-Orbitrap Fusion™ mass spectrometry with peptide HCD fragmentation, we analyzed cytosolic protein extracts of primary human periodontal ligament fibroblasts (hPDLFs) cultured in low and high glucose concentration (8 mM and 25 mM glucose, respectively). Cell cytosolic protein had total MG-H1 residue content of *ca.* 0.42 and 0.72 mmol/mol arg in low and high glucose concentration (*P* < 0.01), respectively, measured by LC-MS/MS analysis of exhaustive enzymatic digests. In Lys C-tryptic digests, Orbitrap Fusion analysis (see Table 3) detected 1077 proteins in both low and high glucose concentration cultures. Thirty proteins were found modified in hPDLF by MG-H1 residues: 10 in hPDLF in low glucose incubation and 20 proteins were detected in high glucose incubations. As a positive control, cell protein extracts were incubated with methylglyoxal to increased MG-H1 content *ca*. 20-fold wherein 173 proteins were detected with MG-H1 modification (unpublished observations) – similar to application of mass spectrometric Orbitrap™ analysis of endothelial cell proteins [40].

## Example of application of mass spectrometry in studies of advanced glycation adducts: glucosepane

Glucosepane is a major glycation-derived quantitative crosslink in physiological systems [115]. Glycation of ribonuclease A with glucose led to the formation of glucosepane cross-links, which were found at residues K41-R39 and K98-R85. The only other intermolecular cross-link observed was the 3-deoxyglucosone-derived 2-ammonio-6-({2-[(4-ammonio-5-oxido-5-oxopentyl) amino]-4-(2, 3,4-trihydroxybutyl)-4,5-dihydro-1H-imidazol-5-ylidene}amino)hexanoate (DODIC) at residues K1-R39. The identity of cross-linked peptides was confirmed by sequencing with tandem mass spectrometry. Recombinant ribonuclease A mutants R39A, R85A, and K91 A were produced and glycated to confirm importance of these sites for cross-linking [116].

## Conclusions/recommendations

Mass spectrometry is often the method of choice for detection and quantitation of glycation adduct content of biological samples where multiplexing for multiple analyte detection is interference-free and addition of further analytes has little incremental cost for analysis. Mass spectrometry proteomics provides for identification of proteins glycated in complex mixtures and concurrent assessment of the effect of glycation on the amounts of all proteins in the sample. Our recommendations for glycation research applications are:

1. Use of stable isotopic dilution analysis LC-MS/MS for detection and quantitation of early and advanced glycation endproducts;
2. use of enzymatic hydrolysis for application to protein samples;
3. immunoassay of glycation adducts be corroborated and referenced to the LC-MS/MS technique where practicable; and
4. use of nanoflow liquid chromatography-Orbitrap™ mass spectrometry with label-free or SILAC approaches for glycated protein identification and quantification in complex mixtures.

In the future there will likely be introduction of stable isotopic dilution analysis LC-MS/MS based quantitation of glycated proteins in to clinical chemistry laboratories.

## Abbreviations

AGC: Automatic gain control
AGE: Advanced glycation endproduct
CID: Collision induced dissociation
CML: N^ε^-Carboxymethyl-lysine
CEL: N^ε^-(1-Carboxyethyl)lysine
DODIC: 2-ammonio-6-({2-[(4-ammonio-5-oxido-5-oxopentyl) amino]-4-(2,3,4-trihydroxybutyl)-4,5-dihydro-1H-imidazol-5-ylidene}amino)hexanoate
ETD: Electron transfer dissociation
FDR: False discovery rate
FL: N^ε^-(1-Deoxy-D-fructos-1-yl)lysine (fructosyl-lysine or fructoselysine).
F3PK: Fructosamine-3-phosphokinase
Glo-1: Glyoxalase 1
HCD: High-energy collisional dissociation
hPDLF: Human periodontal ligament fibroblasts
HSA: Human serum albumin
iTRAQ: Isobaric tags for relative and absolute quantification
LC-MS/MS: Liquid chromatography-tandem mass spectrometry
MALDI-TOF: Matrix-assisted laser desorption-time of flight
MG: Methylglyoxal
MG-H1: N^δ^-(5-hydro-5-methyl-4-imidazolon-2-yl)-ornithine
MG-H2: 2-amino-5-(2-amino-5-hydro-5-methyl-4-imidazolon-1-yl)pentanoic acid
MG-H3: 2-amino-5-(2-amino-4-hydro-4-methyl-5-imidazolon-1-yl)pentanoic acid
MRM: Multiple reaction monitoring
PTM: post-translational modification
SILAC: Stable isotope labelling with amino acids in cell culture
Th: Thomson unit (m/e = 1)
TPCK: Tosylphenylalanylchloromethyl ketone
